# Continuous Estimation of Heart Rate Variability from Electrocardiogram and Photoplethysmogram Signals with Oscillatory Wavelet Pattern Method

**DOI:** 10.3390/s25175455

**Published:** 2025-09-03

**Authors:** Maksim O. Zhuravlev, Anastasiya E. Runnova, Sergei A. Mironov, Julia A. Zhuravleva, Anton R. Kiselev

**Affiliations:** 1 Coordinating Center for Fundamental Research, National Medical Research Center for Therapy and Preventive Medicine, 101990 Moscow, Russiarunnova.ae@staff.sgmu.ru (A.E.R.);; 2 Department of Biophysics and Digital Technologies, Saratov State Medical University, 410012 Saratov, Russia

**Keywords:** oscillatory wavelet patterns, heart rate variability, numerical processing, electrocardiogram, photoplethysmogram

## Abstract

*Objective*: In this paper, we propose a novel approach to heart rate (HR) detection based on the evaluation of oscillatory patterns of continuous wavelet transform as a method of time-frequency analysis. HR detection based on electrocardiogram (ECG) or photoplethysmogram (PPG) signals can be performed using the same technique. *Methods*: The developed approach was tested on ECG (lead V1) and PPG (standard recording on the ring finger of the left hand and differential signal) for 10 min in 40 generally healthy volunteers (aged 26.8 ± 3.22 years). A comparison was made with the traditional HR detection method based on R-peak shape analysis. *Results*: Based on a number of statistical evaluations, the comparison yielded an acceptable degree of agreement between the results of the proposed method and the traditional method (the discrepancy between the results did not exceed 3.41%). The distortion of the signal shape and its noise do not affect the quality of HR detection by the proposed method; so, additional filtering or changes in the implemented algorithm are not required, as demonstrated by processing both the differential PPG signal and the PPG signals recorded during the patient’s walking. *Conclusions*: The proposed method allows obtaining HR information with a higher equidistant sampling frequency and expansion of the information on the frequency composition of HRV.

## 1. Introduction

In recent decades, all countries around the world experienced an increase in the number of deaths caused by chronic and cardiovascular diseases (CVD), which affected the blood vessels and heart (e.g., coronary artery disease). According to the World Health Organization (WHO), CVD is the number one cause of worldwide mortality, with 17.9 million deaths annually [1]. In particular, according to the Russian Society of Cardiology, chronic heart failure claims at least one human life every minute in the Russian Federation [2,3]. Also, according to the European Heart Network report, *European Cardiovascular Disease Statistics 2017*, CVD is responsible for 3.9 million deaths in Europe and over 1.8 million deaths in the European Union (EU) annually. This accounts for 45% of all deaths in Europe and 37% of all deaths in the EU [4].

The main physiological parameters used to evaluate the condition of the cardiovascular system are heart rate (HR) and heart rate variability (HRV). They reflect the autonomic nervous system control and general well-being. HR specifies the number of heartbeats per minute, while HRV indicates the change in time between two consecutive heartbeats. Currently, based on the analysis of HR and HRV, tools for monitoring the physiological condition of the human body, detecting sleep and wakefulness, and assessing attention and other cognitive functions of the patient are implemented [5,6,7]. Systems based on HRV analysis are used in monitoring the attention of drivers and other operators of complex technical equipment [8].

Today, there is a growing interest in wearable devices that both directly monitor the dynamics of cardiovascular system activity and indirectly assess various processes of the body’s functional activity based on the processing of signals from the photoplethysmogram (PPG), ECG, blood pressure, etc. For example, the assessment of the synchronization in the HR, pulse wave, respiration, and other signals is used for automatic monitoring of the patient’s condition in intensive care units (e.g., Hamilton Medical IntelliSync^®^+ technology [9], automatic detection of spontaneous motor activity of patients [10]), for the automatic detection of life-threatening conditions [11], the occurrence of sleep apnea [12,13], etc.

**Figure 1:**
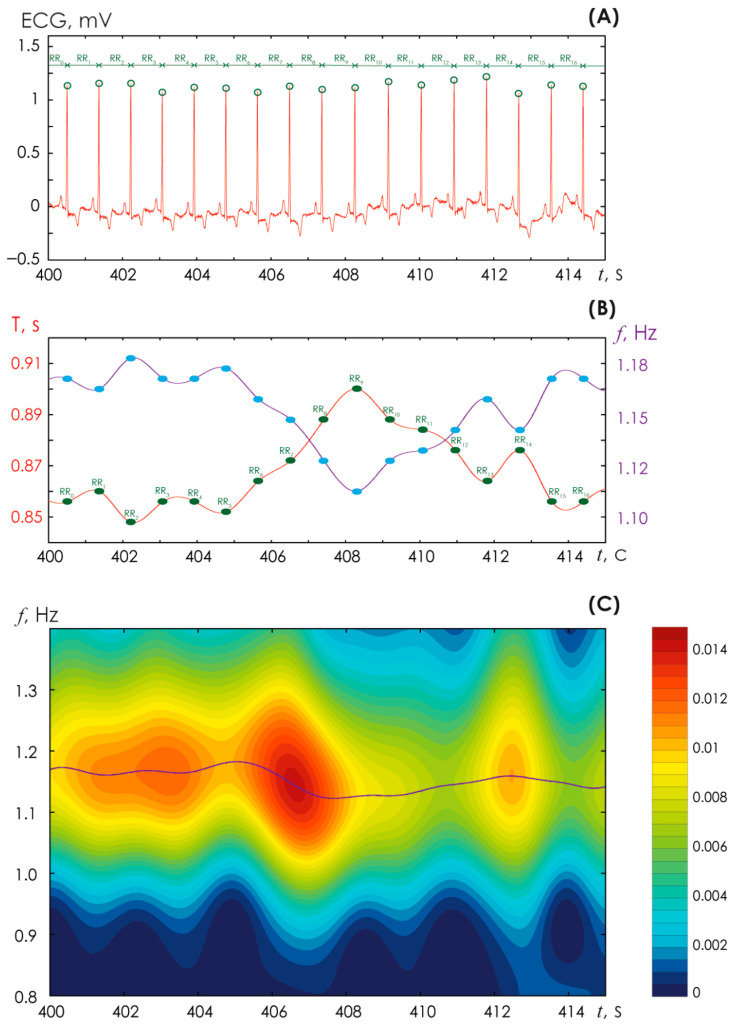
(**A**) Identification of R-peaks on an ECG performed in lead 1 in a generally healthy volunteer. (**B**) Plotting a periodogram of the heart rhythm (shown in red) and a graph of the heart rhythm (shown in purple); the blue points indicate the moments at which the duration of the RR interval is determined. (**C**) Time-frequency surface of the energy of the continuous wavelet transform (CWT) *E*(*f*, *t*_0_) calculated from the ECG fragment presented above in (**A**); a legend for the color designations of the CWT energy amplitudes *E*(*f*, *t*_0_) is shown on the right; the purple line corresponds to the maximum values of the CWT energy and coincides with the purple line in (**B**).

The initial material for analysis of cardiovascular system activity is the RR interval per se (the time elapsing between two consecutive R waves in the ECG). At present, the conventional way to analyze HR is to detect the moment when the highest point R appears in the QRS complex on the ECG. This method, originally developed back in 1985 [14,15], is valued primarily for its simplicity and the possibility of implementation even on the simplest electronic circuits. At the same time, the use of the contour approach can be very difficult in cases of arrhythmia, when the R-peak is deformed, changing its severity and shape from one cardiac cycle to another. Further improvements to this method, such as the transition to considering the first and second derivatives of the signal, as well as the use of certain windowing filtering procedures and averaging [16,17] were aimed primarily at solving these problems. Using multimodal monitoring, this approach has been extended to handle extremely noisy data recorded during movement, for loose electrodes, and in case of other complicating factors [18]. However, the basis of this method remains contour ECG analysis based on identifying the locations of R-maxima in the QRS complexes, as shown in Figure 1A. R-peak location errors can be corrected with the introduction of systems of a higher hierarchical level, for example, within the framework of artificial neural networks and machine learning systems [19] or wavelet analysis methods [20,21]. In the last decade, a method for constructing continuous HRV based on the so-called synchrosqueezed wavelet transform (SWT) has been developed [22].

The resulting RR signal of the HR is nonequidistant, and for further analysis, it is necessary to perform an approximation to transfer to an equidistant signal shape, which will afterwards be used in processing by conventional methods.

PPGs are currently one of the main sources of information about the state of the patient’s cardiovascular system [23]. Contour analysis technology leads to inevitable errors and difficulties in detecting pulse rate on the PPG, especially when patients move [24,25]. Note that the use of SWT technology also requires special adaptation for use in PPG analysis [26].

The development of contemporary methods based on nonlinear dynamics opens up new opportunities for elegant, correct solutions to long-open problems. In particular, one of the recently proposed modifications of the conventional method of time-frequency analysis is the oscillatory pattern approach [27,28,29]. In this paper, we propose a method that to a certain extent eliminates the above-mentioned drawbacks due to the continuous method of HRV detection. The resulting HR sampling frequency when using the proposed method is reduced tenfold compared to the FD sampling frequency of the original ECG recording. For comparison, using the traditional method of R-peak assessment in ECG yields the HR for subsequent analysis with a none-quidistant sampling frequency (on average, close to 1 Hz). The method we developed is adaptive for application to the assessment of ECG and PPG signals. An important factor for using the proposed method is its significant resistance to noise and interference, which are often present on the ECG and, especially, in PPG signals in two situations: when the patient performs physical exercise and when recording signals with uncertified non-professional medical equipment.

## 2. Materials and Methods

### 2.1. Materials

#### 2.1.1. ECG and PPG Recordings of Healthy Volunteers at Rest and While Walking

We recruited 40 generally healthy subjects (9 women and 31 men) among PhD students who participated in our study. The study protocol was approved by the Ethics Committee of the National Medical Research Center for Therapy and Preventive Medicine (Moscow, Russia), and all experimental procedures were performed in accordance with the ethical standards of the Declaration of Helsinki. All subjects were informed about the experimental procedures in detail. Informed consent was obtained from all subjects.

**Figure 2:**
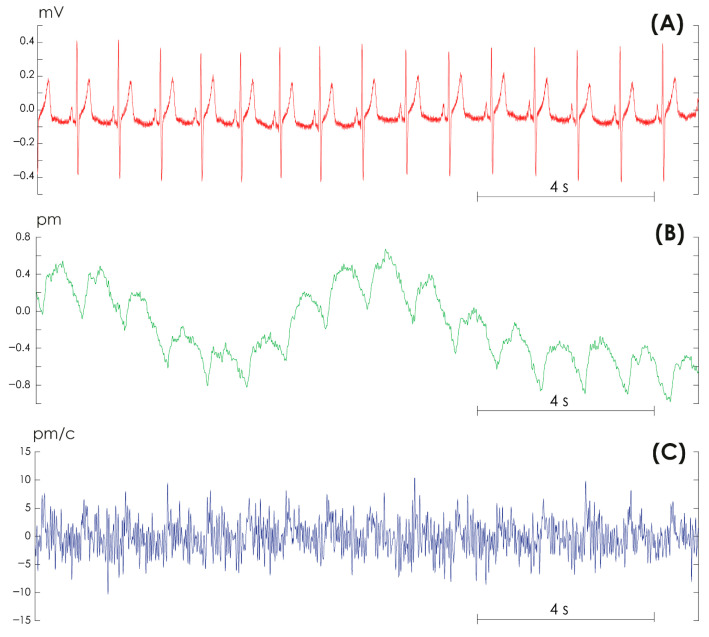
Examples of signals recorded in volunteer #3. (**A**) Electrocardiogram in lead V1. (**B**) Photoplethysmogram from the ring finger of the left hand. (**C**) Differential photoplethysmogram signal.

The mean age of our study subjects was 26.8 ± 3.22 years, and their body mass index was 23.7 ± 3.12 kg/m^2^. For each subject, we simultaneously recorded ECG signals in the V1 lead, PPG from the ring finger of the left hand, and a differential PPG signal (PPGd) for 10 min. Recording was carried out using certified equipment Apnox-10 (Medicom MTD, Taganrog, Russia, https://apnox.com/). The sampling rate for all data was equal to 250 Hz. The reference signal for ECG recording was a triple electrode combined from the upper limbs. Bandpass filters with cutoff frequencies of 0.001 and 30 Hz and a 50 Hz notch filter were applied to all signals. Figure 2 shows fragments of typical ECG, PPG, and PPGd signals recorded from study participant #3. Additionally, a series of ECGs and PPGs were recorded while the volunteers were walking. It is obvious that differential recording of the PPGd signal completely deforms the signal shape, adding noise to it and distorting it, which makes it almost impossible to analyze the signal shape using conventional methods.

An additional six-minute walk test [30] was performed with three volunteers, supplemented by simultaneous recording of ECG and PPG signals. The protocol included 1–2 min of rest, 6 min of walking at the patient’s own pace, 20–25 min of rest, 6 min of walking again, and 5–6 min of rest.

Figure 1A presents the standard procedure for detecting R-peaks on a high-quality ECG signal recorded in lead V1 in a study subject without cardiovascular disease. This procedure is necessary for conventional analysis of the HR. Further on, for each point of the R-peak, the duration of the time interval between the R-peaks, i.e., the period of cardiac contraction (T), can be determined relatively accurately. The latter for the ECG is shown in Figure 1B with a red line. This periodogram signal is not stationary, like any other signal of living systems. Using the known ratio of frequency ν and period T, ν = 1/T, it is possible to construct a graph of HR (blue line in Figure 1B). In this case, the HR, identified by the conventional method of assessing the signal shape, serves as the reference. In relation to it, we can evaluate the effectiveness of the proposed method based on oscillatory patterns.

#### 2.1.2. ECG and PPG Recordings of Elderly Volunteers

From the publicly available MIT-BIH Arrhythmia Database Directory [31], five anonymous patient records were selected with the following numbers: 100 (male, 69 years old), 102 (female, 84 years old), 103 (male), 104 (female, 66 years old), and 107 (male, 63 years old). Each record included 2 ECG channels and RR interval markings from an expert.

### 2.2. Method for Assessing Oscillatory Patterns with Continuous Wavelet Transform

The method for assessing oscillatory patterns was previously developed in [27,32]. At the first stage, the discretized continuous wavelet transform (CWT) was calculated for the ECG/PPG signal [33,34] using the following formula:

(1) $$W\left(1/f,t_{0}\right)=\sqrt{f}\sum_{-\infty }^{\infty }ECG/PPG\left(t\right)\psi^{*}\left(f\left(t-t_{0}\right)\right)∆t,$$

where the symbol * denotes complex conjugate function for the Morlet wavelet basis functions $\psi_{s,t_{0}}(t)$, calculated using Formula (2).

(2) $$\psi (\eta )=\frac{1}{\sqrt[{4}]{\pi }}\exp(2\cdot j\eta \pi )\exp\left(\frac{-\eta^{2}}{2}\right).$$

For each time moment $t_{0}$, the instantaneous frequency distribution of the CWT energy was assessed in the range: *f* ϵ [0.5; 1.5] Hz as follows:

(3) $$E(f,t_{0})=|W\left(f,t_{0}\right){|}^{2}.$$

Figure 1C presents an example of calculating a two-dimensional surface (3) for the ECG signal shown in Figure 1A.

At the second stage, we performed a skeleton assessment of the CWT energy (3) [27,35]. The skeleton assessment involved reduction in the entire time-frequency surface (i.e., of the totality of all bands) of the experimental ECG/PPG signal by examining at each point in time only those frequencies that accounted for the extremums of the surface (3) characterized by zero first and negative second derivatives *E* (*f*, *t*). For the initial moment of time *t*_0_, on the CWT surface *E* (*f*, *t*_0_), a number of local maxima *E_extr_* consisting of *n* points were determined, and frequencies (*f*^0^ = {*sc*_1_, …, *sc_n_*}^0^) were identified at which, at a certain moment of time *t*_0_ these extremes were observed. Further on, for the next moment of time *t*_1_, local maxima of the surface *E* (*f*, *t*_0_) in a certain amount *m* were identified, and the corresponding frequencies *f* ^1^ = {*sc*_1_, …, *sc_m_*}^1^ were identified as well. Now, for each *sc* from the set of frequencies {*sc*_1_, …, *sc_n_*}^0^, a comparison was made with frequencies from the set {*sc*_1_, …, *sc_m_*}^1^. If *sc*^1^ fell into the ϵ-neighborhood of one of the frequencies *sc*^0^, then it was stated that these two frequencies form a single interval of oscillatory activity: {*sc*^0^*, sc*^1^}^p^, if ϵ = 10·∆_D_, where ∆_D_ = (*F_D_*)^−1^ was the sampling period of the original ECG/PPG signal. Such calculations continued further with the repetition of the described procedures. Therefore, for each processed signal (ECG, PPG, PPGd), an array of *N* different patterns {*sc*^0^, *sc*^1^}^p^|_N_ was formed.

## 3. Results

### 3.1. Oscillatory-Pattern Based Algorithm for Detecting Heart Rhythm from ECG/PPG Signals

**Figure 3:**
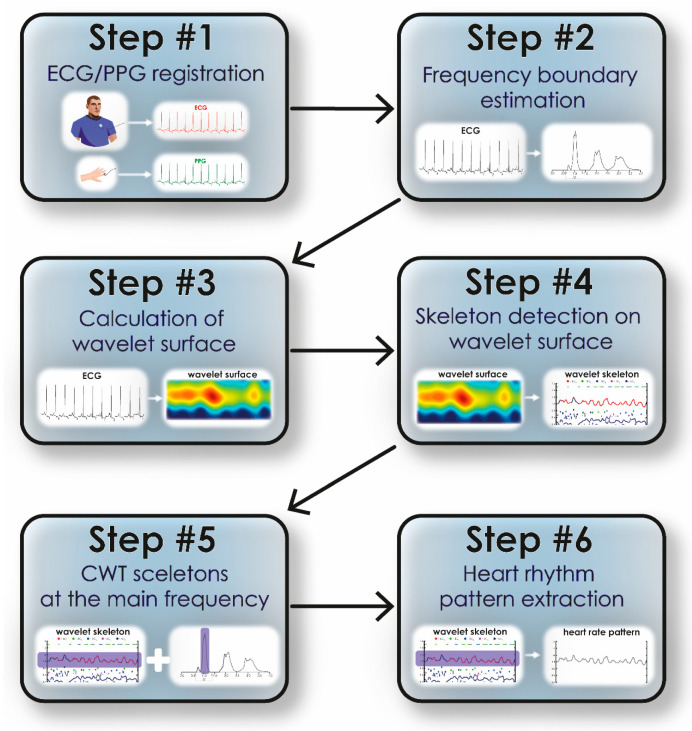
The algorithm of detecting heart rhythm from ECG/PPG signals using the method of CWT oscillatory patterns.

The algorithm for detecting HR from ECG/PPG signals based on oscillatory CWT patterns is presented in Figure 3. After recording ECG/PPG signals (Step #1), a standard fast Fourier transform is performed over the entire signal duration to determine the dominant frequencies (Step #2). At this stage, the fundamental frequency of the signal is identified, *Fmain* ≈ 1 Hz, which accounts for the maximum amplitude of the Fourier spectrum, and the second frequency, which is the double harmonic of the first (*Fmain*)^2^. Then, during Steps #3 and #4, the amplitude *E* (*f*, *t*) (3) is calculated and the skeletons for a given wavelet surface are estimated.

Next, using the main signal frequency calculated during Step #2, we select the band in which oscillatory patterns are detected. The specified band is estimated as [*Fmain* − Fmain*0.2; Fmain + Fmain*0.2]. Similarly, oscillatory patterns are identified at double the frequency (*Fmain*)^2^ in the band [(*Fmain*)^2^ − 0.4*(*Fmain*)^2^; (*Fmain*)^2^ + 0.4*(*Fmain*)^2^]. The identification of the fundamental band is shown in Figure 3 (Step #5). Further on, using the oscillatory pattern method in this frequency band, we plot the heart rate vs. time, as shown in Figure 3 (Step #6). For the method of oscillatory patterns, the following parameters were used: the range ϵ was chosen equal to 0.04 s; the number of calculated skeletons (extrema) did not exceed 25; to characterize frequency changes in the signal, we chose the band [0.8; 1.2] Hz. Finally, based on the described method, the *sc* curve is generated, shown on the time-frequency surface of the CWT energy in purple (Figure 1C).

**Figure 4:**
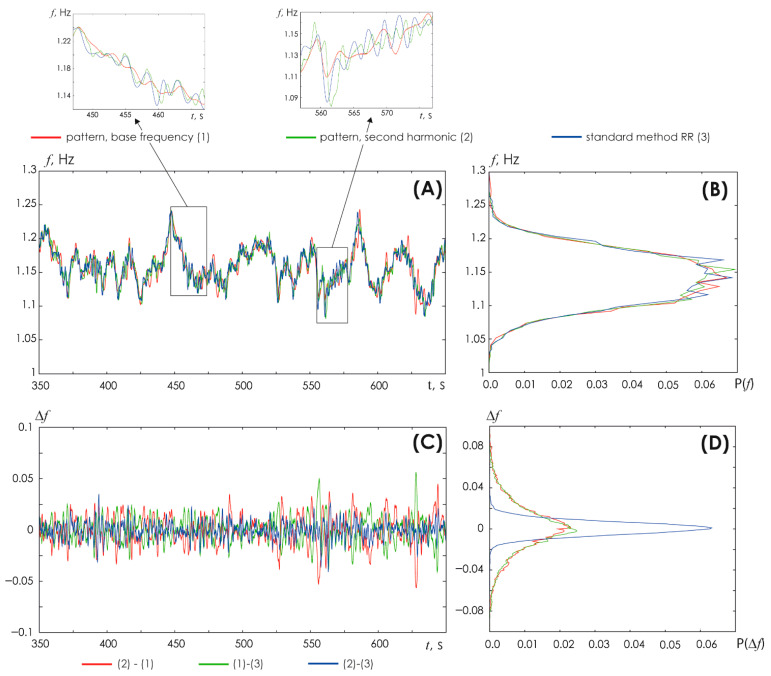
(**A**) Comparison of the results of obtaining HR vs. time by various methods. Red line: conventional method based on the detection of R-peaks on the ECG (1). Green line: method of oscillatory patterns at the fundamental frequency (2). Blue line: the oscillatory pattern method using double the fundamental frequency (3). The insets show enlarged fragments of HR vs. time. (**B**) Probability distribution of HR estimated by various methods: the color scheme of the symbols is identical to (**A**); (**C**) Difference between the results of various methods for determining the HR vs. time. The red line depicts such differences between the method of oscillatory patterns at the fundamental ECG frequency and the conventional method of detecting R-peaks on the ECG: (2)–(1). The green line shows the differences between the conventional method of detecting R-peaks on the ECG and the method of oscillatory patterns at double the fundamental frequency: (1)–(3). The blue line reflects the differences between the oscillatory pattern method at the fundamental frequency and the oscillatory pattern method at double the fundamental frequency: (2)–(3). (**D**) Probability distribution of differences in results obtained by different methods; the color coding is identical to (**C**).

To ensure correct results, Step #6 can be performed for double the fundamental frequency in the signal, i.e., in the band [1.6; 2.4] Hz. We compared the results of determining the HR dependence on time using the conventional method of detecting R-peaks on the ECG (red line in Figure 4) vs. the skeleton method at the fundamental frequency (green line) vs. the skeleton method at double the fundamental frequency of the ECG (blue line in Figure 4A). The insets in this illustration demonstrate enlarged fragments of the time dependence of HR on time. Figure 4B shows the frequency distribution of occurrence of different HR values. It is obvious that different methods yield similar results. However, the method of oscillatory patterns at the fundamental frequency yields the results with a minimal amplitude of changes.

The dependence of the difference between the results of assessing the HR (based on the three used methods) on time is shown in Figure 4C. The red line shows the difference between the HR assessment results by the skeleton method at the fundamental ECG frequency and the conventional method of detecting R-peaks on the ECG vs. time. The green line shows the difference between the HR assessment results by the conventional method of detecting R-peaks on the ECG and the skeleton method at double the fundamental ECG frequency. The blue line shows the difference between the HR assessment results by the skeleton method at the fundamental frequency and the skeleton method at double the fundamental frequency of the ECG. Figure 4D presents the probability distribution of differences between the results yielded by different HR detection methods. Interestingly, minimal differences are demonstrated by the oscillatory pattern method vs. the fundamental and double the fundamental ECG frequencies.

Overall, HR values determined by various methods are very close to each other; however, as the insets in Figure 4A show, the HR identified by the oscillatory pattern method at a fundamental frequency close to 1 Hz is represented by the smoothest waveform of the signal. In the next subsection, we will present a detailed comparison of HR detection results.

### 3.2. Comparison of the Results of HR Detection Using the Method of Oscillatory Patterns for ECG/PPG with the Classical Method of Estimating RR Intervals on ECG

**Figure 5:**
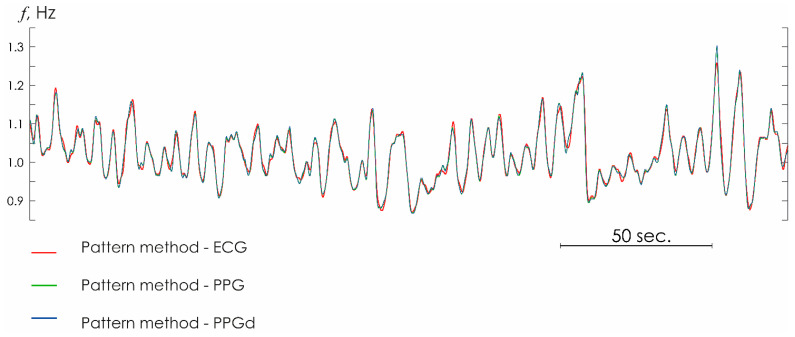
Heart rhythm estimated from ECG (red line), PPG (green line), and PPGd (blue line) signals with the oscillatory pattern method.

Table 1 presents the results of statistical estimates for HR determined by different methods: the conventional method of detecting R-peaks on the ECG and the oscillatory pattern method based on ECG, PPG, and PPGd recordings. Statistical estimates were performed using Formulas (A1)–(A14) in Appendix A. The results of calculations exhibit a good agreement, regardless of the chosen method, not only for the ECG signal but also for PPG recordings. Moreover, the use of the PPGd method with an artificially distorted waveform is no different in terms of obtained results from the results yielded from normal ECG recordings, as clearly demonstrated in Figure 5.

**Figure 6:**
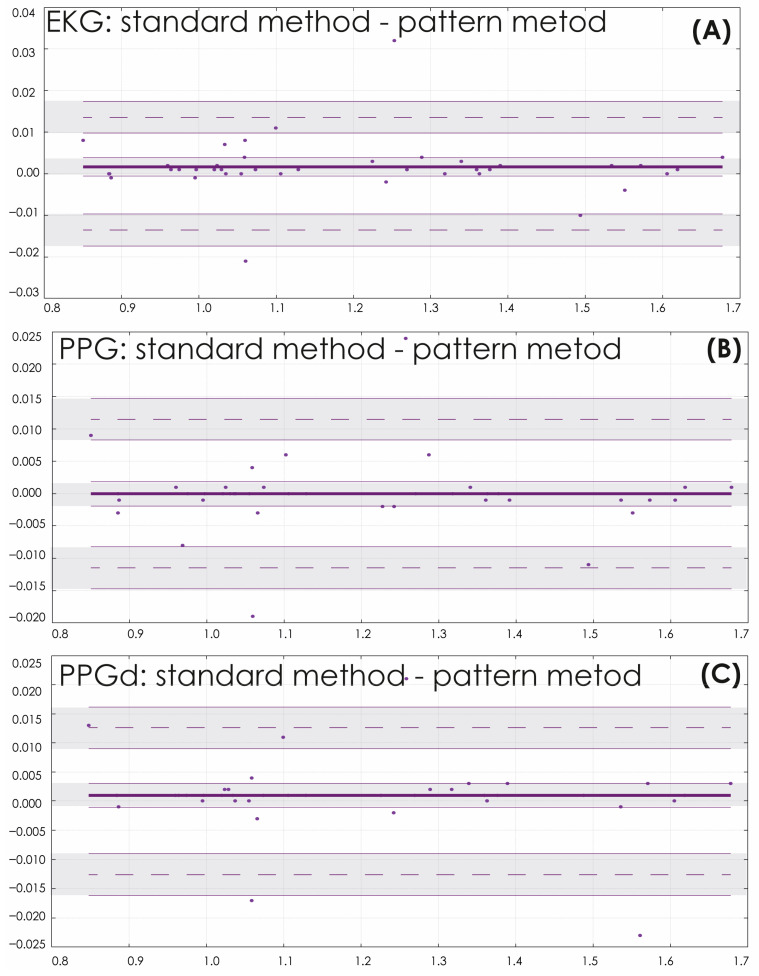
Bland and Altman plots for heart rhythm estimated from ECG (**A**), PPG (**B**), and PPGd (**C**) signals with the standard and oscillatory pattern methods, with the representation of confidence interval limits for mean and agreement limits (shaded areas). The horizontal lines represent: the median (thick solid line), which shows the average difference between the two methods of measuring heart rate, the limits of agreement (thin dotted lines), which indicate the range within which 95% of all differences are lied. The dots on the graph are the differences between the heart rate measurements made by the two methods.

Figure 6 shows the Bland–Altman (B&A) plots, which allow pairwise evaluation of the agreement between quantitative measurements of HR for various signals of the cardiovascular system [36,37]. The average values of the difference between the classical and proposed methods are close to zero (0.0017, −7.5 × 10^−5^, 0.001 when analyzing EKG, PPG, PPGd, respectively.) The concentration of the main difference values in the area of the confidence interval of the central value allows us to cautiously assert the satisfactory quality of processing of the presented ECG and PPG recordings. 5% of the recordings demonstrated an excess of the B&A agreement limits.

The maximum value of the difference between the results yielded by different methods for assessing the HR based on the ECG signal was observed in volunteer #28 and amounted to 3.41%. The maximum differences for PPG signals were found in volunteer #10 (3.53% in the case of regular PPG and 3.77% for PPGd). As expected, differentiation slightly increased the error compared with the original reference ECG recording (by 3.2%).

### 3.3. Demonstration of the Results of HR Detection by the Oscillatory Patterns Method of ECG/PPG Recorded During the Walking of Patients

**Figure 7:**
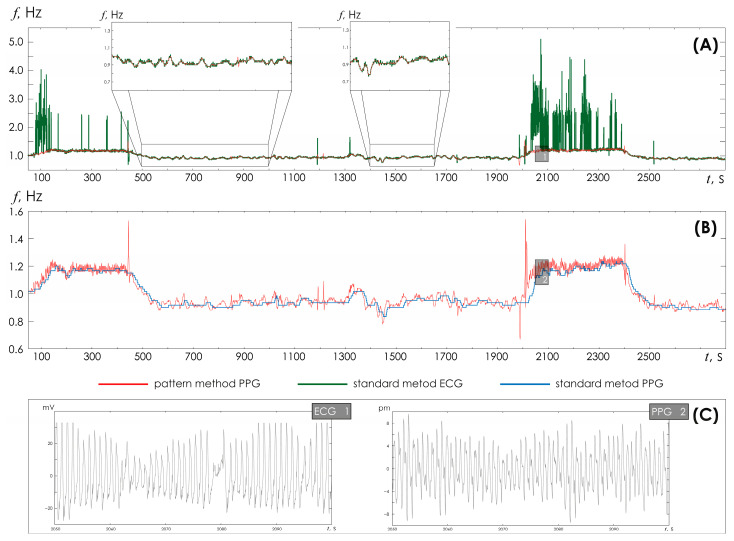
(**A**) Time dependences of heart rate detected in ECG based on the standard method (green line) and the developed method (red line). The inserts show enlarged time fragments of HR recorded at rest. (**B**) Time dependences of heart rate detected in PPG based on the standard method (blue line) and the developed method (red line). (**C**) Fragments of ECG and PPG for the corresponding inserts 1 and 2 from the upper figures.

Figure 7 shows the results of HR assessment performed for records that include walking episodes. Walking episodes are easily distinguished by the increase in HR amplitude. During these periods of walking, significant distortions of the PPG/EKG signals are observed. During periods of rest, the standard technique and the developed HR detection method clearly demonstrate similar results. During moments of movement, the amplitude assessment of R-peak determination does not allow adequately determining changes in heart rate. The standard technique applied to ECG and PPG does not allow determining changes in HR with the same accuracy as the new algorithm proposed in the article allows.

**Figure 8:**
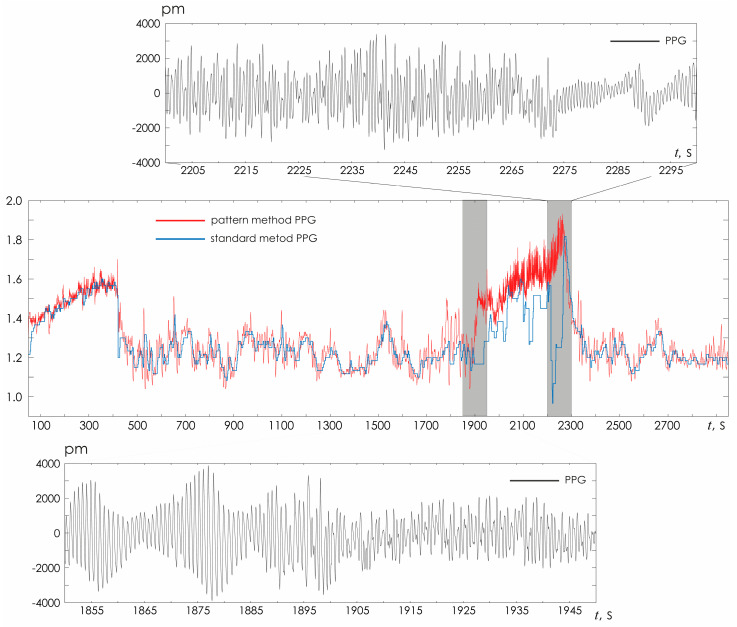
Visual assessment of HRV estimated from PPG with two different methods (standard and oscillatory CWT pattern). The PPG fragments represent example of a signal heavily noisy by movement artifacts during walking.

Figure 8 demonstrates in detail the situation of the PPG signal, noisy by motion artifacts, as shown in the insets above and below. In this case, the standard technique does not allow correct determination of HR during movement. However, the proposed HR detection method retains its functionality, demonstrating the possibility of a detailed study of HR changes over time, which opens up opportunities for correct assessment of the characteristics of HR variability during movement.

### 3.4. Demonstration of the Results of HR Detection by the Oscillatory Patterns Method of ECG Recorded of Elderly Patients (MIT BIH DataBase)

Based on the oscillatory CWT pattern method, the dependence of the heart rate on time was detected for each ECG channel. The result of the HR assessment was compared with the expert markup similar to the previously performed statistical analysis using Formulas (A1)–(A14). The statistical characteristics are presented in Table 2.

**Figure 9:**
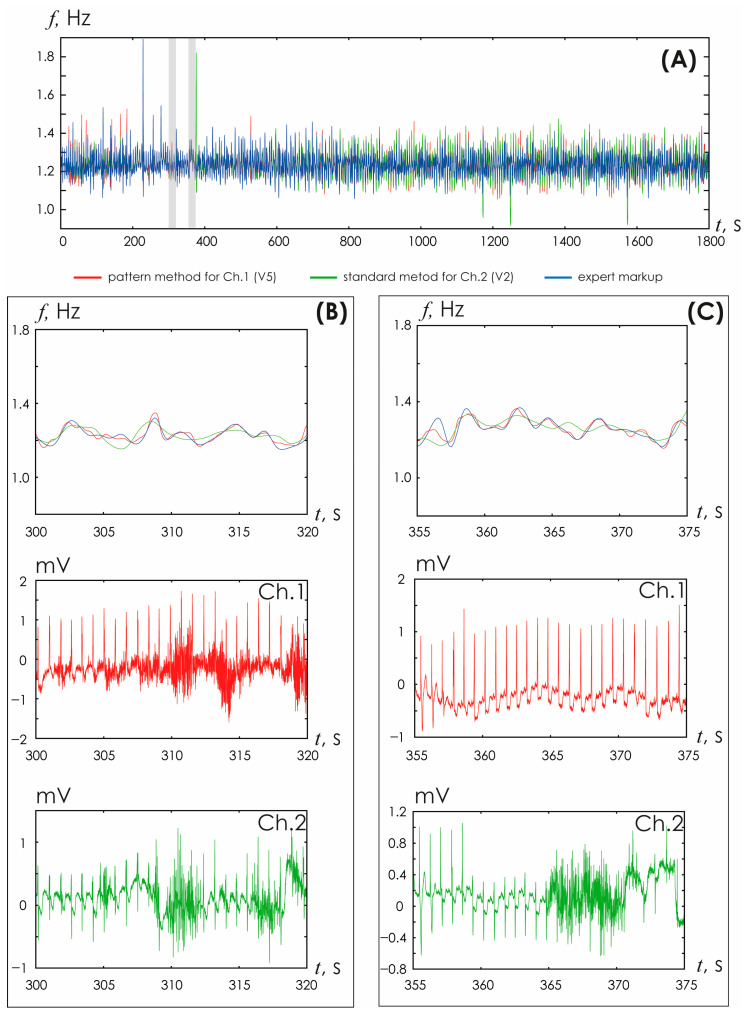
(**A**) Time dependences of heart rate detected in ECG based on the developed method (red line), the standard method (green line), the expert markup (blue line). (**B**,**C**) Enlarged fragments of HR time dependences and ECG recorded in different channels.

Figure 9 shows the dependence of the heart rate on time for different ECG channels of patient # 104. Separate inserts show two ECG sections with noise artifacts, as well as a detailed comparison of HR detection with the expert markup.

The analysis of the presented results demonstrates good correspondence between the heart rate detection based on the oscillatory pattern method and expert marking. In general, the deviations were no more than 5%.

### 3.5. Frequency-Domain Parameters of HR Detected by the Oscillatory Patterns Method of ECG and PPG Signals

For the calculated values of heart rate in the standard LF and HF ranges, the frequency amplitudes were estimated, normalized by the total frequency value in the VLF, LF and HF ranges. The ratio of the frequency amplitudes of slow and fast oscillatory activity, LF/HF, was also estimated. Based on the data obtained, estimates were made of changes in the obtained frequency characteristics for the heart rate estimated in various ways.

**Figure 10:**
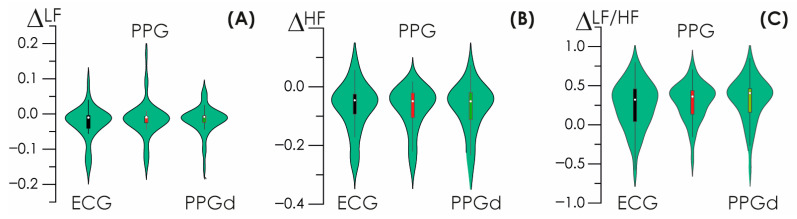
(**A**–**C**) Difference characteristics for LF, HF, and LF/HF frequency characteristics of HR detected based on the standard method and oscillatory CWT method.

These estimates are presented in Figure 10. The distributions shown, box plots, are close to zero values, especially in the HF region of high frequencies. Thus, the implementation of estimates of vegetative modulation, i.e., the activity of the sympathetic and parasympathetic divisions of the nervous system, turns out to be close to those accepted today.

## 4. Discussion

The presented method largely develops the approach presented earlier in [38]. However, Dao et al. considered only the maximum values on the frequency-time surface of the ECG, which allowed for the automatic identification of periods of motor interference. In our study, we implement HR detection by analyzing local maxima on the frequency-time surface, reconstructing the heart rate in a continuous manner.

Traditional methods for assessing signal shapes yield accurate estimates of detecting HR solely for the moments of the appearance of R-peaks on the ECG. Thus, the frequency of ventricular contractions of the heart can be determined with sufficient accuracy only at the detection points of the R-peaks, while at all other time points the HR curve is just the result of a smooth approximation using one or another method (e.g., stepwise interpolation and linear interpolation of different orders [38]). This fact is of particular importance when assessing the HRV. According to the Nyquist–Shannon sampling theorem [39,40,41,42], any function *F(t)*, consisting of frequencies from 0 to *f*_1_, can be transmitted as accurately as desired using discrete points of the Kotelnikov series, following each other in less than *T* = 1/(2·*f*_1_):

(4) $$\tilde{x}\left(t\right)=\sum_{i=-\infty }^{\infty }x\left(i\cdot T\right)\cdot \frac{\sin2\pi f_{1}\left(t-i\cdot T\right)}{2\pi f_{1}\left(t-i\cdot T\right)}.$$

**Figure 11:**
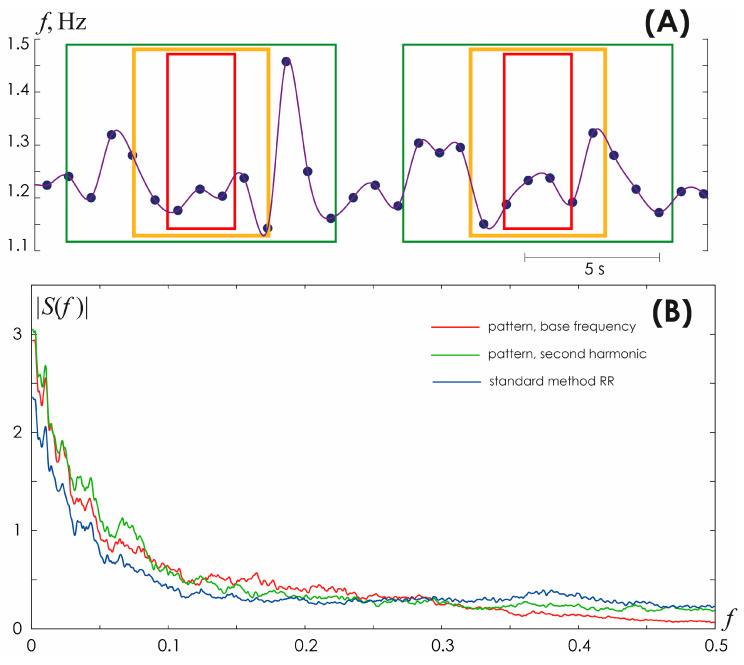
(**A**) Different approaches to approximation of inter beat interval values for a fragment of a heart rhythm periodogram. Green, yellow, and red frames specify the boundaries of the time intervals required to perform frequency analysis of the heart rhythm: green color corresponds to analysis at a frequency of *f* = 0.1 Hz and an oscillation period of 10 s, yellow color corresponds to *f* = 0.2 Hz and an oscillation period of 5 s, red color corresponds to *f* = 0.4 Hz, and oscillation period 2.5 s. (**B**) Spectral composition of HRV determined using the conventional method of detecting R-peaks on the ECG (blue line), the skeleton method using the fundamental frequency based on ECG (red line).

However, the practice of working with ECG presents a more complex situation. First of all, the Nyquist–Shannon sampling theorem provides maximum ratios for ideal conditions, which implies that the spectrum is limited in frequencies and the observation time is infinite. All ECG signals are complex chaotic signals that are limited in time but have an unlimited frequency spectrum. The use of a limited spectrum model and finite observation time leads to errors in reconstructing the continuous signal. Since all terms of Formula (4) become zero at *t* = *i* Δ*t* at all points, with the exception of the term with a serial number of *k* = *i*, then at these points the values of the reconstructed signal coincide with the original values, i.e., at the points of the R-peaks the reconstruction error equals to zero. The error reaches its greatest value within the interval between these readings. Figure 11A demonstrates this pattern for HR signals with accurately identified points solely for R-peaks.

Another cause of the errors is that the spectra of actual time-limited signals do not vanish beyond the cutoff frequency. Although the bulk of the signal energy is located at frequencies from zero to *f*_1_, some fraction of energy occurs at frequencies above the boundary frequency value. The third source of errors is related to the non-ideal parameters of bandpass filters, i.e., functions that approximate RR intervals, as well as numerical errors in determining the coordinates of R-peaks. Therefore, a sufficient number of points for actual complex chaotic signals per oscillation period is typically characterized by the following approximation:

*f*_*D*_ ≈ 2·*λ*·*f*_1_, (5)

where *λ* is some coefficient (as shown empirically for biological signals, it often takes values *λ* ≥ 2.5) [42,43].

Consequently, the evaluation of HR frequency components in the HF band (0.15–0.4 Hz) recommended for consideration when assessing HRV [44] is incorrect, since it is characterized by only 2.5 to 6 points per oscillation period, as clearly demonstrated by the color frames in Figure 11A. These values do not satisfy the condition (5) imposed on the sampling frequency, according to the Nyquist–Shannon sampling theorem for complex chaotic and noisy signals of a biological nature. Such an expansion of information on the frequency composition of HRV may be of interest and useful in constructing AI models for recognizing and/or diagnosing various life-threatening conditions of the body, as, for example, in [45,46,47]. A direct comparison of changes in the frequency spectrum calculated from HR detected using the skeleton method and the conventional method of detecting R-peaks on the ECG is presented in Figure 11B.

In addition, the conventional approach to assessing the shape of ECG/PPG signals allows detecting exclusively the moments of the onset of ventricular contractions (as, for example, this occurs when identifying the R-peaks of QRS complexes). This approach leads to a fundamental simplification of the original ECG signal. Initially, the ECG contains both information about the frequency of ventricular and atrial contractions and information about the conduction time of cardiac excitation in the sinoatrial node and subsequent areas of depolarization. However, all this information is lost or filtered out in a nonlinear way when moving from the analysis of the original reference ECG to the RR interval signal. Using a method based on oscillatory patterns allows detecting the HR with a higher sampling rate while keeping the sampling step equidistant. We believe that the use of the proposed method for analyzing electrophysiological signals of patients with cardiovascular pathologies, such as arrhythmias, coronary artery disease, etc., can provide the doctor with much more information, which requires further research.

The proposed method undoubtedly requires further work in the following areas of research, namely, the development of an approach to real-time analysis based on the assessment of the second harmonic of the fundamental frequency in the electrocardiogram, adaptation of the calculation of classical time domain parameters, as well as nonlinear characteristics of the heart rhythm.

## 5. Conclusions

The article presents a method for detecting HR based on the analysis and assessment of oscillatory patterns. Oscillatory patterns are identified on time-frequency surfaces of continuous wavelet transform using skeleton technology [27,32]. Data obtained from 40 volunteers (simultaneously recorded ECG, PPG, and PPGd in walking and rest) and five patient recordings from the publicly available MIT-BIH Arrhythmia Database Directory demonstrated the high efficacy of the proposed algorithm, as well as its low sensitivity to signal noise.

## Figures and Tables

**Table 1:** Results of statistical estimates for heart rhythm determined by different methods: the conventional method of detecting R-peaks on the ECG, the skeleton method using the fundamental frequency, and the skeleton method using the double the fundamental frequency based on ECG. ECG were recorded in healthy patients at rest.

**#**	$\bar{x^{RR}}$	$\bar{x^{ECG}}$	$\bar{x^{PPG}}$	$\bar{x^{PPG_{d}}}$	$S_{RR}$	$S_{ECG}$	$S_{PPG}$	$S_{PPG_{d}}$	$\bar{{\Delta x}^{RR,ECG}}$	$\bar{{\Delta x}^{RR,PPG}}$	$\bar{{\Delta x}^{RR,PPG_{d}}}$	$\bar{{\Delta x}^{ECG,PPG}}$	$\bar{{\Delta x}^{ECG,PPG_{d}}}$	$\bar{{\Delta x}^{PPG,PPG_{d}}}$
**1**	1.030	1.029	1.030	1.028	0.068	0.068	0.069	0.072	0.013	0.016	0.016	0.007	0.007	0.002
**2**	0.885	0.885	0.885	0.884	0.042	0.044	0.044	0.048	0.018	0.019	0.019	0.006	0.006	0.002
**3**	1.270	1.269	1.270	1.269	0.086	0.087	0.086	0.093	0.038	0.030	0.028	0.013	0.015	0.003
**4**	1.021	1.020	1.021	1.020	0.045	0.045	0.045	0.059	0.044	0.034	0.033	0.035	0.035	0.002
**5**	1.679	1.675	1.678	1.676	0.122	0.124	0.122	0.128	0.029	0.038	0.035	0.031	0.029	0.006
**6**	1.360	1.359	1.361	1.359	0.079	0.081	0.081	0.086	0.014	0.023	0.024	0.016	0.017	0.004
**7**	1.035	1.035	1.035	1.034	0.036	0.037	0.037	0.051	0.030	0.033	0.033	0.008	0.009	0.002
**8**	1.106	1.106	1.106	1.105	0.030	0.027	0.028	0.030	0.008	0.007	0.007	0.006	0.006	0.001
**9**	0.886	0.887	0.887	0.887	0.039	0.035	0.036	0.043	0.025	0.021	0.021	0.013	0.013	0.001
**10**	0.855	0.847	0.846	0.842	0.063	0.049	0.044	0.087	0.057	0.060	0.064	0.020	0.025	0.009
**11**	1.050	1.071	1.069	1.067	0.077	0.088	0.096	0.104	0.065	0.070	0.077	0.064	0.074	0.060
**12**	1.290	1.286	1.284	1.288	0.070	0.085	0.073	0.072	0.018	0.038	0.044	0.038	0.041	0.036
**13**	0.965	0.964	0.973	0.964	0.048	0.051	0.085	0.056	0.024	0.028	0.036	0.011	0.021	0.016
**14**	0.995	0.996	0.996	0.995	0.045	0.043	0.044	0.049	0.024	0.022	0.022	0.012	0.012	0.001
**15**	1.535	1.533	1.536	1.536	0.088	0.090	0.090	0.114	0.023	0.033	0.034	0.025	0.026	0.006
**16**	0.975	0.974	0.975	0.974	0.037	0.036	0.036	0.041	0.015	0.015	0.014	0.003	0.003	0.001
**17**	1.363	1.363	1.363	1.363	0.042	0.042	0.042	0.045	0.010	0.013	0.012	0.008	0.007	0.003
**18**	1.025	1.023	1.024	1.023	0.050	0.051	0.050	0.059	0.029	0.028	0.027	0.010	0.010	0.002
**19**	1.619	1.618	1.618	1.618	0.065	0.058	0.058	0.060	0.036	0.021	0.020	0.036	0.035	0.003
**20**	1.061	1.057	1.057	1.057	0.079	0.032	0.032	0.044	0.052	0.028	0.028	0.056	0.055	0.001
**21**	1.377	1.376	1.377	1.376	0.063	0.061	0.061	0.063	0.017	0.016	0.015	0.009	0.009	0.003
**22**	1.129	1.128	1.129	1.128	0.038	0.038	0.037	0.042	0.010	0.010	0.010	0.004	0.004	0.002
**23**	1.605	1.605	1.606	1.605	0.126	0.124	0.123	0.126	0.033	0.015	0.014	0.025	0.026	0.002
**24**	1.318	1.318	1.318	1.316	0.082	0.082	0.082	0.088	0.035	0.030	0.029	0.008	0.009	0.002
**25**	1.391	1.389	1.392	1.388	0.110	0.114	0.114	0.117	0.019	0.029	0.028	0.016	0.015	0.004
**26**	1.074	1.073	1.073	1.073	0.048	0.048	0.048	0.055	0.023	0.025	0.025	0.005	0.005	0.001
**27**	1.572	1.570	1.573	1.569	0.090	0.100	0.098	0.097	0.045	0.030	0.032	0.031	0.032	0.006
**28**	1.105	1.094	1.099	1.094	0.105	0.099	0.097	0.115	0.075	0.038	0.052	0.057	0.067	0.024
**29**	1.037	1.030	1.037	1.037	0.056	0.041	0.056	0.061	0.019	0.025	0.020	0.014	0.006	0.013
**30**	0.884	0.884	0.887	0.883	0.055	0.057	0.058	0.075	0.030	0.037	0.037	0.016	0.014	0.007
**31**	1.241	1.243	1.243	1.243	0.066	0.055	0.055	0.058	0.027	0.020	0.020	0.020	0.020	0.002
**32**	0.997	0.996	0.997	0.996	0.054	0.053	0.053	0.057	0.019	0.019	0.019	0.010	0.010	0.001
**33**	1.488	1.498	1.499	1.487	0.073	0.157	0.110	0.072	0.038	0.098	0.068	0.108	0.063	0.122
**34**	1.226	1.223	1.228	1.225	0.058	0.065	0.066	0.069	0.044	0.054	0.054	0.027	0.029	0.009
**35**	1.341	1.338	1.340	1.338	0.098	0.089	0.090	0.102	0.038	0.037	0.036	0.027	0.027	0.003
**36**	1.055	1.055	1.055	1.055	0.036	0.036	0.037	0.048	0.012	0.015	0.016	0.010	0.010	0.002
**37**	1.064	1.056	1.067	1.067	0.108	0.098	0.108	0.114	0.043	0.053	0.034	0.043	0.019	0.033
**38**	0.961	0.959	0.960	0.960	0.045	0.045	0.044	0.058	0.026	0.027	0.026	0.006	0.006	0.002
**39**	1.269	1.237	1.245	1.248	0.108	0.116	0.120	0.116	0.051	0.082	0.079	0.071	0.068	0.053
**40**	1.549	1.553	1.552	0.772	0.073	0.099	0.079	0.039	0.052	0.070	0.071	0.047	0.053	0.044

**Table 2:** Results of statistical estimates for heart rhythm determined by different methods: the conventional method of detecting R-peaks on the ECG, the skeleton method using the fundamental frequency, and the skeleton method using double the fundamental frequency based on ECG. ECG were recorded in elderly patients with cardiac arrhythmia.

**No.**	$\bar{x^{RR}}$	$\bar{x^{ECG_{1}}}$	$\bar{x^{ECG_{2}}}$	$S_{RR}$	$S_{ECG_{1}}$	$S_{ECG_{2}}$	$\bar{{\Delta x}^{RR,ECG_{1}}}$	$\bar{{\Delta x}^{RR,ECG_{2}}}$	$\bar{{\Delta x}^{ECG_{1},ECG_{2}}}$
**100**	1.259	1.259	1.259	0.082	0.052	0.051	0.039	0.038	0.009
**102**	1.211	1.210	1.212	0.048	0.044	0.028	0.040	0.030	0.028
**103**	1.162	1.162	1.163	0.050	0.042	0.042	0.030	0.030	0.005
**104**	1.235	1.234	1.238	0.055	0.053	0.062	0.045	0.051	0.030
**107**	1.184	1.189	1.184	0.063	0.052	0.036	0.042	0.032	0.025

## Data Availability

The data presented in this study are available on request from the corresponding author.

## Appendix A. Evaluation of the Quality of Heart Rate Determination Using the Oscillatory Pattern Method

We assessed the following comparative characteristics of HR using various methods: viz., the conventional assessment of RR intervals by the method of the first derivative identification and the method of oscillatory patterns. In particular, we calculated the means of $\bar{x^{...}}$ for HR values obtained by different methods based on ECG and PPG signals as follows:

(A1) $$\bar{x^{RR}}=\frac{1}{n}\sum_{i=1}^{n}x_{i}^{RR},$$

(A2) $$\bar{x^{ECG}}=\frac{1}{n}\sum_{i=1}^{n}x_{i}^{ECG},$$

(A3) $$\bar{x^{PPG}}=\frac{1}{n}\sum_{i=1}^{n}x_{i}^{PPG},$$

(A4) $$\bar{x^{{PPG}_{d}}}=\frac{1}{n}\sum_{i=1}^{n}x_{i}^{{PPG}_{d}}.$$

here and below, we use the following notation: *i*, the serial number of the HR sampling step; *n*, the number of points in the original time series at which the HR was determined; the *RR* index correspond to HR detection based on the conventional method of identifying R-peaks; *ECG*, *PPG*, and *PPGd* indices correspond to HR detection by the oscillatory pattern method based on the signals of ECG in the lead V1, the PPG recorded on the left ring finger, and the PPGd of the patient, respectively.

Estimates of the standard deviation $S_{\ldots }$ were calculated for HR values obtained by various methods based on ECG and PPG signals as follows:

(A5) $$S_{RR}=\sqrt{\frac{1}{n}\sum_{i=1}^{n}{\left(x_{i}^{RR}-\bar{x^{RR}}\right)}^{2}},$$

(A6) $$S_{ECG}=\sqrt{\frac{1}{n}\sum_{i=1}^{n}{\left(x_{i}^{ECG}-\bar{x^{ECG}}\right)}^{2}},$$

(A7) $$S_{PPG}=\sqrt{\frac{1}{n}\sum_{i=1}^{n}{\left(x_{i}^{PPG}-\bar{x^{PPG}}\right)}^{2}},$$

(A8) $$S_{{PPG}_{d}}=\sqrt{\frac{1}{n}\sum_{i=1}^{n}{\left(x_{i}^{{PPG}_{d}}-\bar{x^{{PPG}_{d}}}\right)}^{2}}$$

The difference characteristics $\bar{{∆x}^{\ldots ,\ldots }}$ for HR values identified by various methods from ECG and PPG data were also estimated as follows:

(A9) $$\bar{{∆x}^{RR,ECG}}=\frac{1}{n}\sum_{i=1}^{n}{(x}_{i}^{RR}-x_{i}^{ECG}),$$

(A10) $$\bar{{∆x}^{RR,PPG}}=\frac{1}{n}\sum_{i=1}^{n}{(x}_{i}^{RR}-x_{i}^{PPG}),$$

(A11) $$\bar{{∆x}^{RR,{PPG}_{d}}}=\frac{1}{n}\sum_{i=1}^{n}{(x}_{i}^{RR}-x_{i}^{{PPG}_{d}}),$$

(A12) $$\bar{{∆x}^{ECG,PPG}}=\frac{1}{n}\sum_{i=1}^{n}{(x}_{i}^{ECG}-x_{i}^{PPG}),$$

(A13) $$\bar{{∆x}^{ECG,{PPG}_{d}}}=\frac{1}{n}\sum_{i=1}^{n}{(x}_{i}^{ECG}-x_{i}^{{PPG}_{d}}),$$

(A14) $$\bar{{∆x}^{PPG,{PPG}_{d}}}=\frac{1}{n}\sum_{i=1}^{n}{(x}_{i}^{PPG}-x_{i}^{{PPG}_{d}}).$$
